# Nematobacterial Complexes and Insect Hosts: Different Weapons for the Same War

**DOI:** 10.3390/insects9030117

**Published:** 2018-09-11

**Authors:** Maurizio Francesco Brivio, Maristella Mastore

**Affiliations:** Laboratory of Comparative Immunology and Parasitology, Department of Theoretical and Applied Sciences, University of Insubria, 21100 Varese, Italy; maristella.mastore@uninsubria.it

**Keywords:** entomopathogenic nematodes, innate immunity, insect host, *Steinernema feltiae*, *Steinernema carpocapsae*, *Xenorhabdus bovienii*, *Xenorhabdus nematophila*, host-parasite

## Abstract

Entomopathogenic nematodes (EPNs) are widely used as biological control agents against insect pests, the efficacy of these organisms strongly depends on the balance between the parasitic strategies and the immune response of the host. This review summarizes roles and relationships between insect hosts and two well-known EPN species, *Steinernema feltiae* and *Steinernema carpocapsae* and outlines the main mechanisms of immune recognition and defense of insects. Analyzing information and findings about these EPNs, it is clear that these two species use shared immunosuppression strategies, mainly mediated by their symbiotic bacteria, but there are differences in both the mechanism of evasion and interference of the two nematodes with the insect host immune pathways. Based on published data, *S. feltiae* takes advantage of the cross reaction between its body surface and some host functional proteins, to inhibit defensive processes; otherwise, secretion/excretion products from *S. carpocapsae* seem to be the main nematode components responsible for the host immunosuppression.

## 1. Introduction

Nematodes are one of the most abundant groups of animals on earth [1], and thanks to their small size, their resistant cuticle and their ability to adapt to severe environmental changes, they have colonized a wide range of habitats including vertebrates’ and invertebrates’ bodies, so nematodes may be free-living or parasitic [2,3]. The latter are usually considered pests because they cause important diseases in animals and humans, and due to their economic impact on many agricultural products.

A small but significant number of parasitic nematodes, called entomopathogenic nematodes (EPNs), are of considerable interest because they possess various features that could allow them to be used as biological control agents for pest insects [4,5,6]. EPNs must meet criteria to be considered good candidates for biological control: they should target environmental pests and insect vectors, and be able to kill, sterilize, or hamper the development of their insect targets. Insect-parasitic nematodes that possess optimal features as bio-insecticides belong to the families Steinernematidae and Heterorhabditidae (Nematoda, Rhabditidae). Steinernematidae and Heterorhabditidae are not closely related phylogenetically, but through convergent evolution, they have similar life histories [7].

The natural host range of *Steinernema* spp. and *Heterorhabditis* spp. can be defined as the range of insects which indigenous nematode populations use for their propagation. A distinction has however to be made between the range of insect species being susceptible to nematodes in the laboratory, the range of hosts successfully controlled by inundative release of nematodes (field host range) and the range of insects on which a naturally occurring nematode population propagates. The latter, called the natural host range, is poorly defined, thought an overview on this subject has been proposed by Peters [8].

*Steinernematidae* and *Heterorhabditidae* use different method of parasitization that can be defined as static (ambushing) or active (cruising); *Steinernema* spp. are usually considered as static, since they wait for host proximity, but there are many examples of mixed foraging strategies by which they combine ambushing and cruising approaches to reach the host [9,10]. *Heterorhabditis* spp. actively approach their host, even if we cannot exclude that there are species not yet described, that behave like ambush foragers.

Regarding reproductive strategies, *Steinernema* spp. are mostly gonochoristic but there are hermaphroditic species such as *S. hermaphroditum* [11], *Heterorhabditis* spp. are commonly considered as hermaphroditic, although, as demonstrated by Chaudhuri et al. [12] *Rhabditis* sp. SB347, a nematode with sexual polymorphism, produces males, females and hermaphrodites.

Most of the nematodes in these families differ from other Rhabditidae by having a species-specific mutualistic relationship with bacteria: *Xenorhabdus*, associated with Steinernematidae [13,14], and *Photorhabdus*, associated with Heterorhabditidae [15,16,17,18,19]. However, non-symbiotic bacteria associated with different EPNs have been described in the literature [20], these microorganisms have been found either in the gut or in other body regions [21]. The role of these bacteria has not been completely clarified, even if they sometimes show a pathogenicity towards the host and resistance to antibiotics produced by the symbionts of the nematode [22,23]. Some exceptions have been reviewed by Dillman et al. [24] who stated, about some newly described associations, that nematode-bacterium partnerships should not be considered entomopathogens if they do not explicitly fulfill the requirements to be classified as EPNs, e.g., the heritability of their bacterial associations.

**Figure 1:**
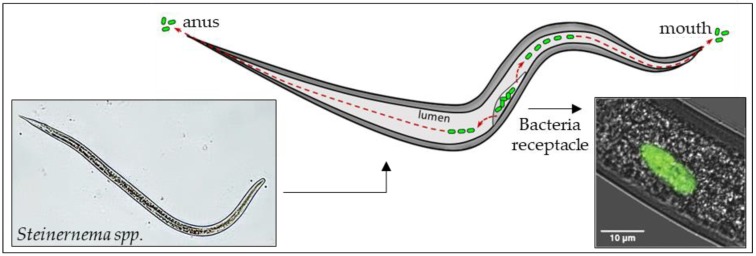
EPNs symbiotic bacteria (*Xenorhabdus* spp.) live in a monoxenic area, or in differentiated vesicles of the anterior part of intestine, modified as a bacterial receptacle. Micrograph at right shows fluorescent GFP-transformed bacteria inside *Steinernema* spp. intestine, (image at right, courtesy of J. Chaston, from [25]).

The symbiotic bacteria (Figure 1) contribute to the nematodes’ ability to kill the host; they establish suitable conditions for nematode reproduction, providing nutrients and inhibiting the growth of other microorganisms in the insect host, by the release of antimicrobial compounds.

At the same time, the nematode acts as a vector for the symbiotic bacteria, and, by interacting early with the host immune system, it prepares a favorable environment for its symbionts. The symbiotic relationship is essential for the efficiency of the biocontrol and it enables nematodes to exploit a diverse array of insect hosts [26,27].

In general, endoparasites penetrate invertebrate hosts by overcoming the first line of defense, consisting of the exoskeleton and mucosal tissues of the external openings and once they reach the hemocoel cavity, they must elude the host’s recognition system and/or depress immune effector processes [28].

**Figure 2:**
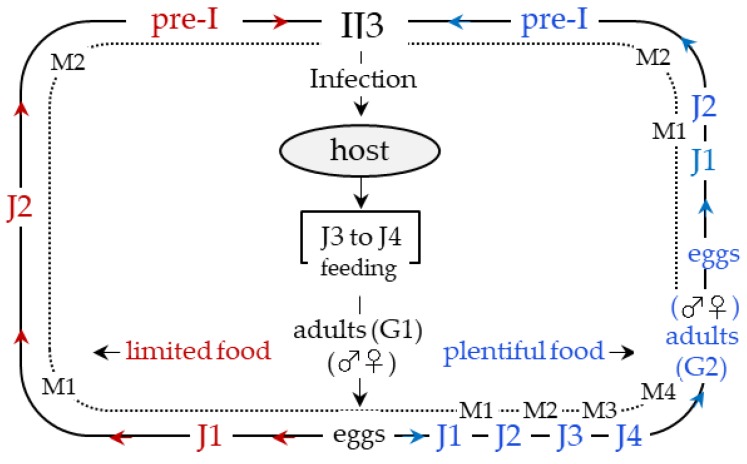
EPNs life cycle. After infection of the host, the infective juvenile stage (IJ3) develop to J4 and to adults (G1), and after mating the eggs develop to J1. If food is scarce, they molt in succession to J2 and pre-I (pre-infective stage juvenile), which will become infective (IJ3). Otherwise, in the presence of abundant food, nematodes molt in succession to the fourth stage (J4), and to adults (G2), and after mating eggs develop into J1, J2, pre-I and finally to IJ3. At the IJ3 stage nematodes search for new hosts to infect. M: Molt; J: Juvenile stage; G: Generation.

It is generally accepted that, to survive, a parasite must reach an ideal equilibrium with its host; however, in the case of EPNs, the parasite must kill its host and though this could seems a disadvantage, because it reduces the number of hosts, it is an essential condition for EPNs, since they use the host corpse as an environment for the development of their offspring. Thus, EPNs can be considered as parasitoids whose behavior is characterized by the lethal nature of their interaction with their host. It is very important to be aware that EPNs are nematode-bacteria complexes and that the success of their parasitic interaction (Figure 2) with the infected hosts is based on the synergism between the parasite itself and its symbiotic bacteria. In addition, to gain an in depth understanding of the host-parasite relationship that is established by EPNs, it is pivotal to summarize processes and components of the insect immune system that are involved in recognition and neutralization of invaders.

## 2. An Overview of the Insect Immune System: Sensing and Recognition of Non-Self

Insects possess a potent innate immune system by which they attempt to resist microbial infections and parasitic invasions [29]. However, this may not always be effective because some invaders have developed sophisticated survival mechanisms to live and complete their life cycles within the host body. Insects, and invertebrates in general, can discriminate between self and non-self by means of a powerful innate immune system [30,31], and consequently, endoparasites must overcome the host immune defenses to complete their life cycles [32,33].

The innate immune system is characterized by the absence of adaptive immune recognition receptors, although pattern-recognition receptors (PRRs) are used to interact specifically with a broad range of foreign antigenic compounds, commonly named pathogen-associated molecular patterns (PAMPs). The interaction between PAMPs and PRRs is a crucial step in the discriminatory processes of innate immunity that usually precedes the effector mechanisms responsible for the elimination of non-self [34,35,36,37]. Innate immunity is common to all metazoans and serves as a first-line defense against foreign antigens. Innate immune responses are not specific to a particular antigen in the way that the adaptive immune responses are, its hallmarks are the recognition of non-self by germ line-encoded non-rearranging receptors and rapid effector mechanisms that involve several cell-mediated and humoral processes [38,39].

**Figure 3:**
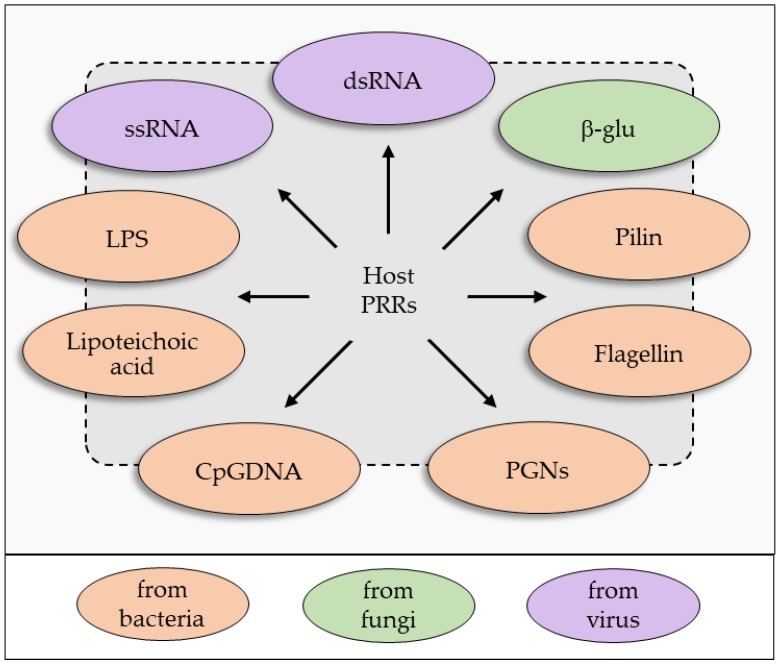
PAMPs from invaders are recognized by free, or cell-associated host receptors (PRRs). Typical PAMPs are: bacterial lipopolysaccharide (LPS), bacterial peptidoglycans (PGNs), β-glucans from fungi (β-glu), lipoteichoic acid from Gram positive bacteria, bacterial flagellin and pilin, nucleic acid variants such as single or double-stranded RNA (ssRNA, dsRNA) and unmethylated CpG motifs (CpGDNA).

Insect responses involve both cellular and humoral defense mechanisms, and all these processes are triggered by free and membrane-bound PRRs capable of specifically binding to PAMPs [35]. PAMPs are molecules that are common to groups of pathogens, these can be referred to as small molecular motifs conserved within a class of microbes, and they are recognized by free or cell-associated receptors (PRRs) in all animal species. The prototypical PAMPs (Figure 3) could be molecules secreted or derived from the surface of bacteria and fungi, or nucleic acid variants normally associated with viruses [40,41,42,43,44,45,46,47].

### 2.1. Humoral Immune Recognition

In insects, the presence of non-self (microorganisms or metazoans) can selectively stimulate several immune reactions and sometimes lead to the differential expression of effector genes, but any immune reaction is preceded by the interaction of PRRs with PAMPs and thus of the subsequent triggering of defensive reactions [35,48,49].

Many proteins that act as PRRs have been identified in the hemolymph of various insect species. The roles of PRRs have been exhaustively discussed by Kanost and co-workers [36], who described peptidoglycan-receptor proteins (PGRPs), β-glucan receptors (β-GRPs) and immuno-related oligosaccharides-receptors, known as immulectins (IMLs), in *Manduca sexta* (Lepidoptera). In recent years, the central role of the PGRP family has been highlighted; the sensing of peptidoglycan molecules by these receptors seems to be essential to activate different pathways of innate immunity. Glucan-binding proteins (GNBPs) specific for DAP-type and Lys-type peptidoglycans from Gram-negative and Gram-positive bacteria, respectively, [50] are involved in *Toll* and *Imd* pathways and they stimulate the synthesis of antimicrobial peptides. Further roles for soluble PGRPs have been suggested, for example they can function as opsonic factors, bactericidal factors and modulators of the prophenoloxidase activating system (proPO-AS) [51,52]. A PGRP was isolated as a 19 kDa protein in *M. sexta* hemolymph, and its mRNA, found in fat bodies, seems to be either constitutive or inducible a few hours after injection of bacteria. *Manduca* PGRP is responsible for the activation of several immunological pathways, without enhancing the activity of host proPO-AS [53,54]. Two β-GRPs are present in the hemolymph (β-GRP-1 and β-GRP-2) which bind β-1,3 glucans and lipoteichoic acid, thus agglutinating both Gram-negative and Gram-positive bacteria and triggering host proPO-AS [55]; the mRNAs for these proteins are constitutively present in fat body cells (β-GRP-1 mRNA) or up-regulated following infection of bacteria or yeast (β-GRP-2 mRNA).

A third family of PRRs found in *M. sexta* belongs to the C-type lectins (calcium-dependent lectins); these proteins contain at least a 110–130 carbohydrate-recognition domain that interacts with free proteins or lipid-conjugated oligosaccharides [56,57]. Four immulectins (IMLs) were isolated and characterized in bacteria-challenged larval hemolymph, all are involved in binding of lipopolysaccharides (LPS). For example, IML-1 and IML-2, when complexed with LPS, modulate the *Manduca* proPO-AS [58,59].

In 1997, Dunphy and Halwani [60] isolated two LPS-binding proteins (LBPs), named LBP-1 (17.2 kDa) and LBP-2 (26 kDa), from *G. mellonella* naïve larvae; the authors reported that these soluble receptors are specific for the bacterial surface and seem to act as endotoxin detoxifiers, thus protecting hemocytes from damage. Both LBPs are specific for the lipid A region of LPS and LBP-1 acts as an activator of proPO-AS. Wiesner and co-workers [61] isolated and described a hemolymph protein called Apolipophorin-III (ApoLp-III), with a molecular mass of 17 kDa; ApoLp-III has been identified as an immune-stimulating factor in lepidopteran insects. The immune-stimulating capacity of ApoLp-III is surprising, since this protein is known to play an important role in lipid transport in flying insects. Injection of natively purified, as well as of recombinant ApoLp-III [62], from *G. mellonella* into the hemocoel of untreated larvae led to a strong increase of antibacterial activity within the hemolymph [63]; the induced antibacterial activity reached the same intensity as that which can be maximally provoked by injecting bacteria. Thus, ApoLp-III represents a further endogenous mediator which is involved in the regulation of insect immune responses [64]. Therefore, the discriminatory phase of innate immunity seems to be based on a few receptors that have the ability to recognize a large number of foreign molecular patterns.

### 2.2. Cellular Immune Recognition

In insects, hemocytes freely circulate in the hemolymph, or are localized in specific regions of the body (e.g., fat body cells). Hemocytes play a role in several defensive functions against foreign targets, and in general, they need to be activated by the presence of PAMPs and/or by endogenous soluble factors. This activation is often mediated by specific membrane receptors that are able to recognize and bind their co-receptors. Following this interaction, hemocytes become stimulated and initiate complex mechanisms such as intracellular signal transduction that culminates in the activation of specific immune genes [65], or initiates defense mechanisms such as phagocytosis, encapsulation, nodulation and proPO-AS release [66,67,68]. Furthermore, non-opsonin-mediated phagocytosis is an ancient form of pathogen recognition that is mediated by the direct interaction of hemocyte membrane receptors with pathogen surface molecules. In *Drosophila*, the scavenger receptor *Eater* is expressed by hemocytes [69], and its N *terminus* is responsible for direct binding to microbes. Therefore this membrane protein plays a key role in the removal of Gram-positive and Gram-negative bacteria [69].

**Figure 4:**
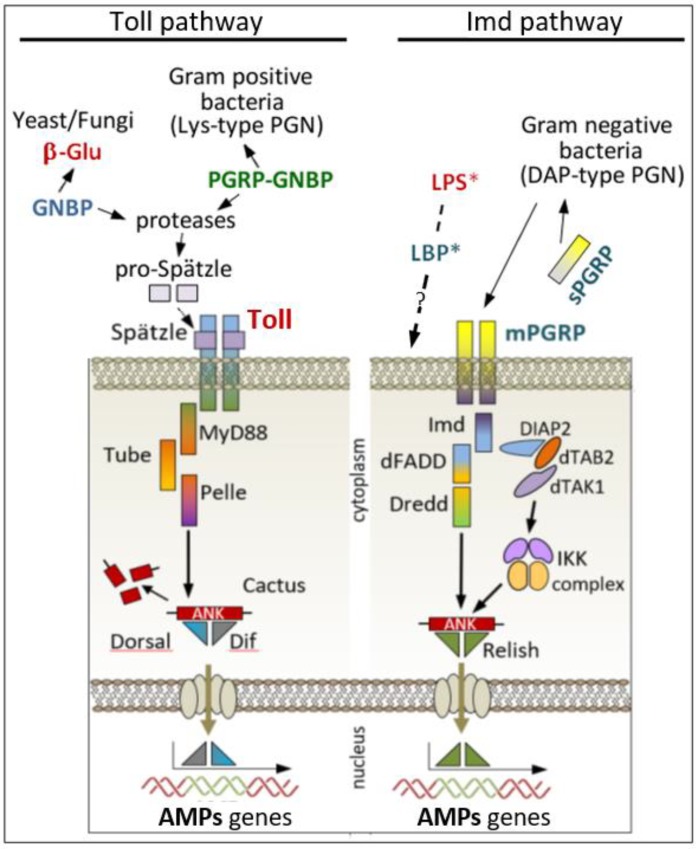
Cellular PRRs: *Toll* and *Imd* pathways. (**Left**) a schematic view of fungine and Gram-positive bacteria stimulation of *Toll* pathway; the intracellular signal transduction involves adaptor proteins as MyD88, Tube and the serine/threonine protein kinase Pelle. Upon activation, *Toll* receptor-adaptor complex signals to a latent transcriptional factor, belonging to the NF-kB-Rel family, NF-kB factor is normally complexed to an inhibitor protein (IkB-like) named Cactus. The *Toll* signaling induces the degradation of Cactus and dissociation from Rel family proteins (Dorsal and Dif). Dif, a member of NF-kB-like proteins, seems to be the main transactivator factor responsible for antifungal and anti-Gram positive bacterial defenses. Dif translocates into the nucleus and switches on many AMPs (antimicrobial peptides) genes, probably several hundred, that in concert contribute to challenge microbial infection. (**Right**) the *Imd* pathway stimulated by Gram negative bacteria. The *Imd* gene encodes a 25 kDa protein with a *death domain*, the protein acts upstream of an adaptor protein named FADD; furthermore, for a full activation, a caspase-8 like protein is required (Dredd). Dredd can directly activate REL protein (Relish). Finally, Rel domain after cleavage, moves to the nucleus and activates AMPs genes. Otherwise, Relish can be phosphorylated by IKK signaling complex, which is itself activated by Tak1 (a MAP kinase) interacting downstream of *Imd*/FADD.

One of the most intriguing discoveries of recent years was that of the evolutionarily conserved *Toll* and *Imd* pathways (Figure 4), found in *Drosophila* [70,71] and humans [34]. *Toll* is a transmembrane receptor with an extracellular domain rich in leucine repeats, whereas the intracellular region has a significant homology with the corresponding region of the Interleukin-1 receptor (IL-1R); this part of the receptor is referred to as the TIR domain (*Toll*-IL-1R). *Toll* receptors are expressed either on fat bodies or epithelial cell membranes after stimulation by fungi or Gram-positive bacteria [72]. The interaction of *Toll* receptor with its extracellular ligand Spätzle (Spätzle is turned into its active form when PRR-PAMP interactions led to activation of serine protease) leads to activation of an intracellular cascade, which culminates in the activation of antimicrobial peptides-coding genes (Figure 4, left panel). A second pathway (Figure 4, right panel), the immune deficiency pathway (*Imd*) is activated when Gram-negative bacteria infect the host [73]. While the *Imd* intracellular transduction pathway is well known [72] and several cellular sensing receptor(s) have been identified [74], the complete picture is far from being elucidated. Evidence seems to indicate that PGRPs (peptidoglycan receptor proteins) may be key receptors for peptidoglycans of Gram-negative bacteria [75,76,77].

Insect mutants for *Toll* and *Imd* genes were found to be differentially susceptible to fungal and bacterial infection [78,79,80]. In these mutants, the *Imd* and *Toll* pathways do not appear to share any intermediate components and mediate differential expression of AMP-encoding genes via distinct NF-kB-like transcription factors [71]. So far, *Toll* and *Imd* pathway cellular receptors could be considered as the main receptors involved in PAMP sensing at a cellular level [81]. Their role is not limited to AMP gene activation [82,83], but, from an evolutionary standpoint, they represent a further confirmation of the ancient origin of innate immunity [84], and a study model that may be used to progress our understanding of a unifying concept of an innate immune response common to all metazoans.

### 2.3. Effector Processes

**Figure 5:**
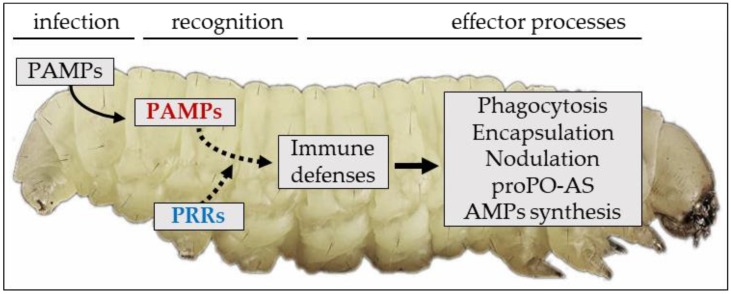
After entry, PAMPs interact with hemolymph and cell-membrane receptors (PRRs) triggering humoral and cellular defenses of the insect host.

Insects defend themselves from challenge by microorganisms or parasites using a combination of humoral and cellular responses (Figure 5) by means of effector processes triggered following interaction between their receptors and foreign molecules.

However, the distinction between these two processes is rather artificial, as infection elicits a complex of responses that are mostly coordinated and cooperative to fight potential invaders. In addition, immunocompetent cells, even if mainly responsible for cell-mediated immunity, can release soluble compounds belonging to the humoral factor pool. However, to simplify these responses is useful when considering cellular and humoral immunity as separate topics.

### 2.4. Humoral Defence

The circulating fluid of insects is called hemolymph, and unlike blood, it is not confined within vessels but freely distributed in an open circulatory system. Hemolymph plays a key role in transport and storage of nutrients and hormones. In addition, a large pool of soluble factors is synthesized and released by hemocytes and functions as immune molecules that cooperate in both recognition and elimination of invaders. Among these, components such as lysozyme, phenoloxidase and AMPs, contribute to the elimination of non-self.

### 2.5. Melanization

**Figure 6:**
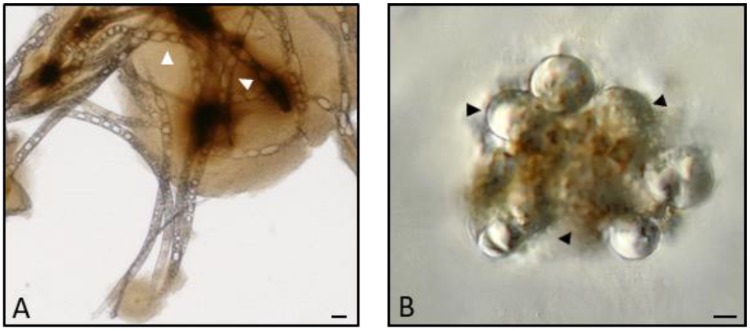
(**A**): Free-living nematodes (*Panagrolaimus rigidus*) incubated with cell-free hemolymph, arrowheads indicate melanin-trapped worms. (**B**): Humoral encapsulation of synthetic microbeads. Bars = 50 μm. (unpublished images by Brivio [86]).

An important process among insect humoral responses is melanization which is responsible for the formation of melanin coats surrounding foreign bodies. This defense mechanism, called humoral encapsulation [67,85], is efficacious and usually faster than cell-mediated processes. The penetration of metazoan or bacteria can elicit the activity of the proPO-AS, the main function of which is the synthesis of melanin in the process of humoral encapsulation (Figure 6A). This enzymatic cascade is extremely reactive, since it can also be activated in the presence of inert materials such as charged microbeads (Figure 6B).

The melanization reaction, which is a common response to the presence of non-self in invertebrates (especially arthropods), is due to the activity of an oxidoreductase called phenoloxidase. This molecule is the terminal enzyme of a complex system of proteases, proteases inhibitors (serpins) and humoral PRRs, constituting the proPO-AS. proPO-AS is proposed to be a system of recognition of non-self, since the conversion of prophenoloxidase to active enzyme can be easily achieved by foreign molecules such as LPS, PGNs and β-1,3-glucans [87].

**Figure 7:**
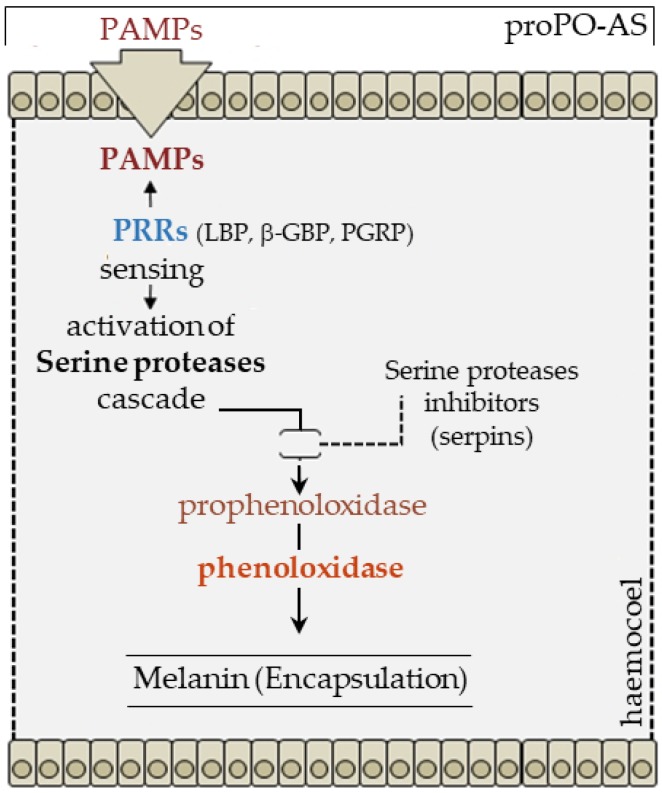
Insect proPO activating system is strongly elicited by the presence of PAMPs, prophenoloxidase zymogen, after PRRs-PAMPs interaction, is activated by serine proteases cascades; physiological negative control (serpins) provide the downregulation of the enzyme activity to turn-off the system when not required. The system is responsible of humoral melanization (melanin encapsulation) of foreign targets.

Prophenoloxidase is converted into its active form by a limited proteolysis, and when activated, phenoloxidase can oxidize phenols into quinones that in turn autocatalyze into melanin [87,88]. proPO-AS is a key element in the recognition of foreign bodies and it is also involved in production of opsonic factors; it is now considered to represent an integral component of the insect immune system [89,90,91]. Several hemolymph molecules, functioning as PRRs, are involved in proPO-AS (Figure 7), among them β-1,3-glucans-binding proteins (β-GBP), LBPs and PGRPs, seem to play key roles as receptors triggering the protease cascade [87,92,93].

### 2.6. Antimicrobial Peptides and Bacterial Clearance

Insect antimicrobial peptides (AMPs) are synthesized in fat body tissues (comparable with the vertebrate liver) and released into the hemolymph during a systemic response against pathogens. The sensing of foreign bodies culminates in the synthesis, ex novo, of AMPs. Thus, these are considered to be inducible factors. Their occurrence is consequent to the activation of immune genes mediated by evolutionarily conserved *Toll*/*Imd* pathways present in both vertebrates and invertebrates.

AMPs are strong cationic, heat stable and amphipathic molecules that have a variable amino acid composition, length (30–60 aa) and structure.

Despite their structural variety, AMPs are able to affect a large number of microorganisms [94,95]; for example, positively charged peptides interact directly with the anionic moiety of bacterial membranes (e.g., LPS, lipoteichoic and teichoic acids), inducing an increase in membrane permeability that leads to rapid cell death [95,96]. When fat body cells are stimulated by the presence of foreign compounds, intracellular pathways (*Toll*/*Imd*) become active and AMP gene transcripts led to the synthesis of a pool of antimicrobial peptides active against Gram-negative and Gram-positive bacteria, fungi and yeast [97]. Following table (Table 1) summarizes some characteristics of AMPs isolated from insects.

**Figure 8:**
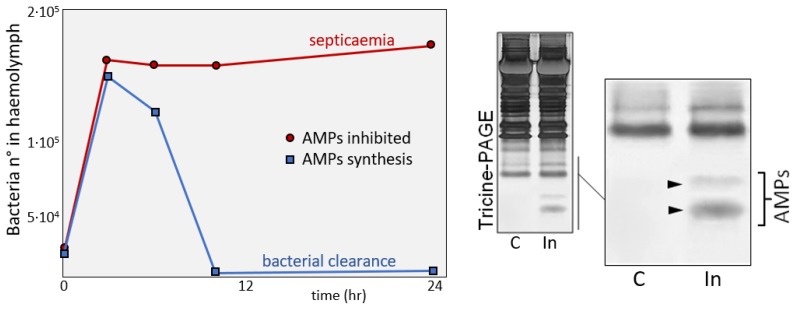
(**Left**) effects of AMPs synthesis after bacterial infection; insect larvae when inoculated with bacteria show an efficient bacterial clearance by means of newly synthesized AMPs (blue line). If the infection was carried out in the presence of inhibitors of protein synthesis (red line), AMPs were absent, and the larva showed symptoms of septicemia disease. (**Right**) Electrophoretic analysis of hemolymph samples from not infected (C) and infected (In) *G. mellonella* larvae. Arrowheads indicate the presence of two low molecular mass bands in the hemolymph from immunized larvae, (from [99]).

The activity of AMPs against bacterial walls is supported by the action of lysozyme, a constitutive enzyme that hydrolyses bacterial wall peptidoglycans [98]. Lysozyme is described as a constitutive factor whose concentration is related to the increase of bacteria in the host body. Physiologically, AMPs production (Figure 8) and upregulation of lysozyme synthesis reflect the requirement of insects to fight infection and to avoid the risk of septicemia.

### 2.7. Cellular Defenses

Many studies on insect immunity have been performed to identify immunocompetent cells and understand their functions when in the presence of non-self invaders [65,100].

Hemocytes are circulating cells that play a key role in preserving host integrity, and they are involved in cellular mechanisms such as phagocytosis, nodule formation, encapsulation, cell-mediated melanization and synthesis of antimicrobial peptides [36,101,102]. Therefore, hemocytes are directly responsible for the elimination of foreign bodies such as bacteria, fungi, nematodes etc.

**Figure 9:**
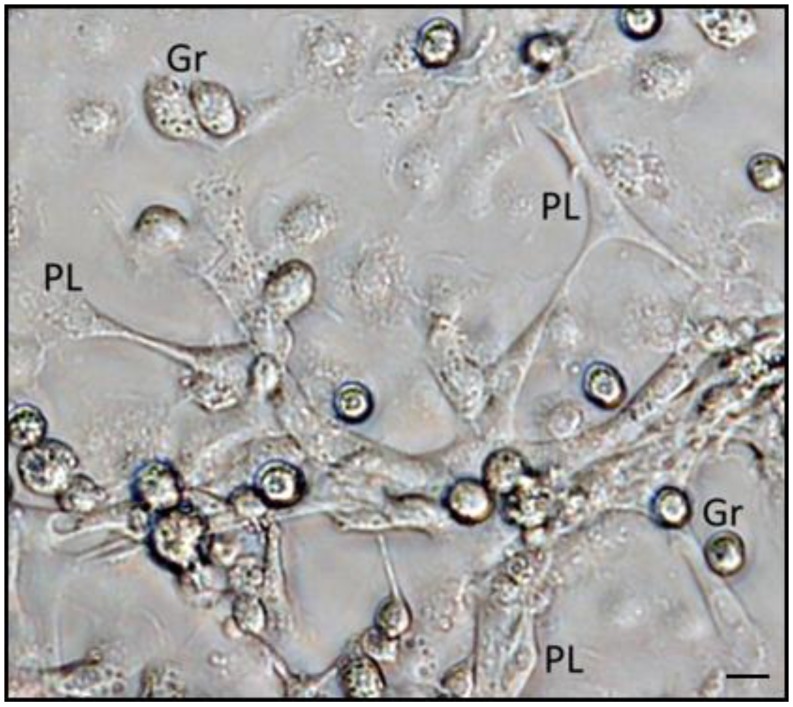
Nomarski Interference Contrast (NIC) micrograph of a culture of *G. mellonella* hemocytes: Adherent plasmatocytes (PL) and many reacted and not-reacted granulocytes (Gr) are observable. Bar = 50 μm, (Unpublished micrograph by Brivio [86]).

Circulating cells within hemolymph consist of several populations that are classified according to morphological, histochemical, immunocytochemical and functional features [103,104,105]. Common types of hemocytes, described in Lepidoptera (Figure 9), are the pro-hemocytes, granulocytes, plasmatocytes, spherulocytes and oenocytoids [106,107,108,109].

Classification of insect hemolymphatic cells is somewhat different in Dipterans, where lamellocytes and cells with crystalline inclusions (crystal cells) have been described in addition to plasmatocytes [100,110]. During insect embryogenesis, a fraction of cells develops from the head or from the dorsal mesoderm; the tissue continues to produce hemocytes during larval or nymphal stages via division of stem cells in the hemopoietic organs and/or by continued division of hemocytes already present in the hemolymph [111,112,113].

Pro-hemocytes, are an immature form of hemocytes, they seem to represent stem cells able to differentiate into one or more cell types. In the larval stage, granulocytes and plasmatocytes are the most abundant cell type in Lepidoptera, and are known to be capable to recognize, adhere to and spread on foreign surfaces, otherwise not-adherent cells, spherulocytes, oenocytoids and pro-hemocytes represent a small fraction of the circulating hemocyte population [114].

When foreign organisms breach the outer physical and chemical barriers of an insect and penetrate its hemocoel cavity, the cellular components of the host immune system may be rapidly mobilized and a struggle for survival ensues.

### 2.8. Phagocytosis

The primary response of hemocytes to small particles, such as bacteria, yeast, or protozoa, is phagocytosis, a process that can be envisioned as a specialized form of receptor-mediated endocytosis. The process of phagocytosis, from insects to mammals, appears to be very similar [115]. In both cases, binding of opsonic ligands to the surface of the particle occurs, which is then followed by receptor-mediated recognition and subsequent activation of signaling pathways resulting in the internalization of the foreign body [116,117].

**Figure 10:**
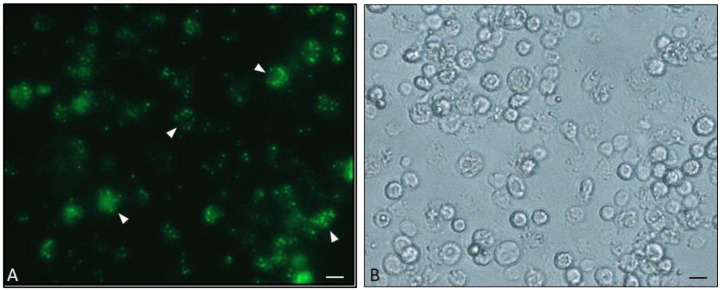
An example of in vitro phagocytosis. (**A**): Fluorescein Isothiocyanate (FITC)-conjugated *Escherichia coli* cells (arrowheads) are engulfed by lepidopteran hemocytes (he). (**B**): brightfield image. Bars = 50 μm, (unpublished micrographs by Brivio [86]).

In insects, both granulocytes and plasmatocytes have been reported to be able to phagocytize (Figure 10); the efficacy of phagocytosis depends on the structure of the surface of the foreign organism and on the involved hemocytes.

In addition, the presence of microbial factors, such as glucans, PGNs or LPS, can increase the phagocytic rate of hemocytes. Moreover, lectins (or lectin-like molecules) can play a role in the opsonization of non-self; in general, phagocytosis can be enhanced by the interaction between foreign sugars (free or conjugated oligosaccharides) and hemolymph sugar-binding proteins [118].

Finally, the process may be stimulated by the same components released after proPO-AS activation [119]. In the presence of many bacterial cells (or large amounts of free LPS) or fungi, hemocytes degranulate releasing humoral factors that form aggregates, called nodules [68], this process leads to the entrapping of foreign cells. Such nodular aggregates may adhere to host tissues and larger nodules may eventually be encapsulated by the hemocytes [120].

### 2.9. Encapsulation

**Figure 11:**
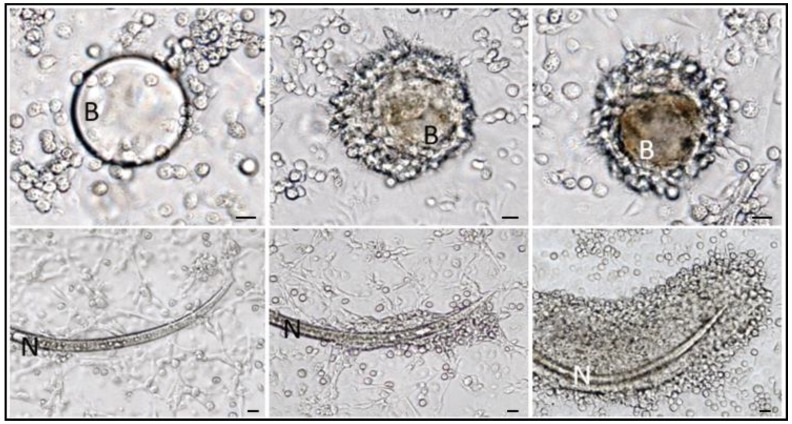
*G. mellonella* hemocytes forming capsule around abiotic materials and free-living nematodes. (**Upper**) panels show encapsulation steps from 1 to 8 h after incubation of cultured hemocytes with synthetic beads (B); in the right micrograph is evident the melanin deposition around the bead inside the inner cell layer. In (**lower**) panels the same experiment was carried out with free-living nematodes *P. rigidus* (N). In both assays the progressive formation of multi-layered cellular capsules is observable. Bars = 50 μm (from [121]).

When protozoan, metazoan parasites, eggs or larvae and foreign invading organisms or abiotic particles are too large to be phagocytized, they can be encapsulated by multiple layers of hemocytes. These hemocytes can produce a coat of melanin in the late stage of the process and this process is named cellular encapsulation (Figure 11).

The formation and growth of cellular capsules mainly requires two cell types: granulocytes and plasmatocytes [100,113]. Usually, the formation of a capsule begins within 30 min from the entry of a foreign body and the early steps involve granulocytes and humoral pattern-recognition receptors (PRRs) as opsonic factors. In sequence, granulocytes release chemotactic components, called plasmatocyte-spreading peptides (PSP), that attract plasmatocytes and increase their adhesive properties [122,123,124].

As mentioned above, humoral factors may also be involved in encapsulation. In particular, in the early recognition and binding of PAMPs, different studies have demonstrated that humoral PRRs are needed to stimulate the aggregation of plasmatocytes on the surface of the target or to the earlier layers of the granulocyte capsule [67,101]. The cooperation between these immunocompetent cells results in a multicellular layered thick capsule that segregates the foreign organism, avoiding trophic exchanges with the host body environment. Moreover, the toxic effects of melanin, which is present inside the inner layers of the capsule, may contribute to kill the entrapped organism [125,126,127,128].

## 3. An Overview of Parasites’ Strategies

Since the effectiveness of insect immune defenses has been exhaustively described, how can EPNs overcome/avoid the host defensive processes? The two main strategies by which EPNs avoid and counteract the host immune defenses are molecular mimicry processes and immune suppression, these strategies can be particularly effective when they penetrate young hosts with a low level of immune competence; however, EPNs are commonly able to invade both adults and dead hosts [129,130,131].

In general, mimicry processes can be realized by the synthesis of molecules that are antigenically related to the host (usually named self-proteins), and are exposed on the parasite body surface [132,133,134]. Otherwise, mimicry could be a form of disguise based on the acquisition of host molecular compounds or tissues which overlay the parasite body surface [32,135,136]. Moreover, depression of host defenses is usually achieved thanks to the excretion/secretion of various compounds that interfere with and neutralize many effector processes elicited by the host in response to infection [137,138].

Many studies have described the strategies implemented by EPNs and their symbiotic bacteria to survive and reproduce inside their insect hosts and it is commonly accepted that the main strategies are immune evasion and immunosuppression.

**Figure 12:**
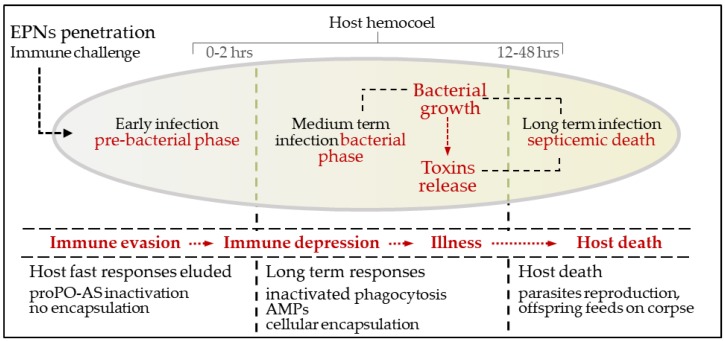
EPNs: time course of EPNs infection, modulation of immune response and host death.

In the early phase following infection (0 to 2 h), to overcome host defenses, the worms use a type of mimicry to become unrecognizable to proPO-AS and to the immunocompetent hemocytes; in subsequent phases, both nematodes and symbiotic bacteria use active strategies aimed to depress humoral and cellular responses, based on the release of toxins, inhibitors and proteases. The nematocomplexes behave like Trojan horses, carrying and releasing their symbionts inside the host’s hemocoelic cavity; thus, *Xenorhabdus*, when in the hemolymph, need to be unrecognized in the early stages of infection. The following scheme (Figure 12) describes the sequence of events following the penetration of EPNs in the host.

In this review we describe the behavior of two EPNs belonging to the same genus, *Steinernema feltiae* and *Steinernema carpocapsae*, that are associated with the symbiont bacteria *Xenorhabdus bovienii* and *Xenorhabdus nematophila*, respectively. These EPNs, by means of different elusive strategies, achieve a successful life cycle inside their insect hosts.

### 3.1. S. feltiae: Host Immunomodulation

In this section we describe the effects induced by *S. feltiae* after penetration of the hemocoel of *G. mellonella*. The main features of this EPN are the effects of the elusive processes by which the worm interferes with the host immune responses; in these processes the body surface of nematodes (epicuticle/cuticle) plays a central role and its involvement has been described by various authors [139,140]. A general model of the immune-evasive role of the nematode cuticle was proposed by Blaxter et al. [141], despite the fact that different species show differences in their molecular architecture and properties. In addition, the epicuticular outer layer can be modified in composition and organization, and in relation to the host internal milieu, EPNs molt multiple times during their life cycle. Each time they molt, they change their body surface, building a new cuticle and epicuticle. Cuticles of various parasitic nematodes, with other surface and secreted molecules, participate in immune evasion and suppression of host defenses [142,143].

A specific role for the *S. feltiae* epicuticle has been suggested by Dunphy and Webster [144]. The authors described the lack of encapsulation by *G. mellonella* hemocytes and presumed that the lipidic moiety present in the epicuticle of the nematode could be responsible for this process. Indeed, they observed that the treatment of the cuticle with lipases caused the loss of its elusive properties; they supposed that the alteration of the cuticular lipids led to the unmasking of discriminable antigens.

Unlike *S. carpocapsae*, *S. feltiae* does not seem to use secretion processes to induce host immunosuppression, thus if depressive phenomena are observable, before bacteria release, they can reasonably be attributed to the parasite body surface.

As discussed above, some papers proposed an intriguing hypothesis by which some unidentified molecular components, present on the body surface of the EPN, could play a role not only in the mimicry processes, but also in the depressive phenomena that are observed in the early stages after the penetration of the nematocomplex. Relying on those assumptions, knowledge of the relationship between the body surface of the parasite and the immune responses of the insect host have been extensively investigated [145]; authors defined a temporal interval that allowed them to exclude the action of the symbiont bacteria (before their release), i.e., a short period in which the observed immunodepressive processes could reasonably be attributed to the nematode itself.

**Figure 13:**
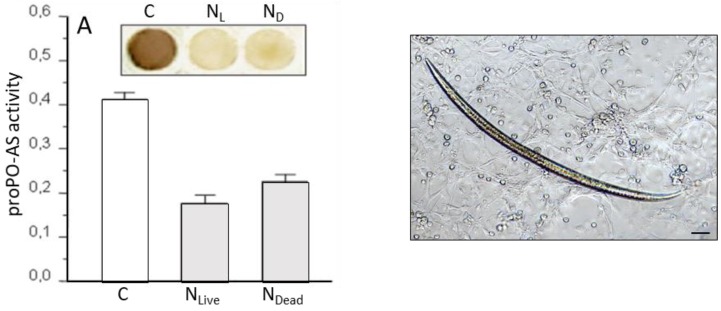
(**Left**) Effects of the presence of live and dead *S. feltiae* inside host hemocoel 30 min after infection, the presence of parasites induces a marked inhibition of phenoloxidase activity; the upper box shows the lack of melanin due to the presence of live or dead nematodes (N_L_, N_D_). (**Right**) Co-incubation of live parasites with *G. mellonella* cultured hemocytes, showing the absence of cellular encapsulation. Bar = 100 μm, (from [146]).

Preliminary observation, obtained with live or dead whole parasites, demonstrated that the worm was unrecognized, did not trigger the proPO-AS (Figure 13, left) and it was not encapsulated by host cells (Figure 13, right); so it was clear that the body surface was able to avoid host immune recognition by means of an elusive strategy. Isolated cuticles from *S. feltiae* obtained by an improved technique, also confirmed their elusive properties [145].

**Figure 14:**
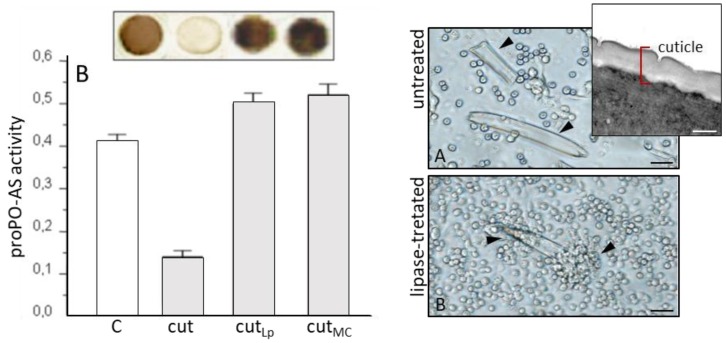
(**Left**) inhibitory effects induced by isolated *S. feltiae* cuticles (cut) compared to the basal PO activity (C). The damage of cuticle lipids by lipase treatments (cut_Lp)_ or methanol-chloroform extractions (cut_MC_) resulted in a marked activation of the host proPO-AS. The upper box shows the lack of melanin synthesis in the insect hemolymph due to the presence of isolated cuticles (cut), melanin was evident when the hemolymph was incubated with treated cuticles (cut_Lp_ and cut_MC_). (**Right**) co-incubation of untreated and lipase-treated cuticles with *G. mellonella* hemocytes; untreated cuticles are not recognized and encapsulated; instead, when they were treated with lipases, migration of hemocytes and encapsulation were observed. Inset: TEM micrograph of the *S. feltiae* cuticle/epicuticle in peripheral region of body. Black bars = 100 μm; white bar = 500 nm, (from [146]).

The use of cuticles has allowed to exclude any effects due to nematode secretions or to their symbiotic bacteria (Figure 14), and to avoid the use of axenic nematodes which could be physiologically altered because of the absence of their symbionts. Thus, in the presence of the parasite cuticle, a drastic immunodepressive effect was observed, and the inhibition of the host proPO-AS was evident either after in vivo cuticle injection or in vitro co-incubation with hemolymph (Figure 14, left, cut). The molecular architecture of the cuticle and epicuticle is critical to preserve immunosuppressive effects because chemical alterations cause a loss of their properties (Figure 14, left). Confirming Dunphy and Webster suggestions [144], Brivio et al. [146] demonstrated a key role of the surface lipidic moiety, since its removal or alteration by lipases (cut_Lp_) or methanol-chloroform (cut_MC_) treatments, made the cuticles detectable by the host immune system.

As observed with whole nematocomplexes, isolated cuticles were not recognized by host hemocytes (Figure 14, right, untreated) while lipases treatments induced the loss of their elusive properties (Figure 14, right, lipase-treated).

**Figure 15:**
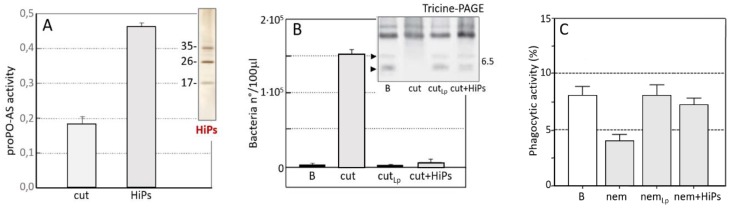
Effects of *S. feltiae* and its cuticles on the host immune processes. (**A**): The activity of host proPO-AS, inhibited by the presence of *S. feltiae* cuticles, was restored by the addition of host-interacting proteins (HiPs) eluted from the parasite surface after co-incubation with host hemolymph. Inset: an SDS-PAGE of HiPs. (**B**): The presence of cuticles interferes with AMPs synthesis, since a marked growth of bacteria in the hemolymph from cuticle-injected larvae is observable (cut). Treatments with lipase (cut_Lp_) or addition of HiPs (cut + HiPs), result in a marked bacterial clearance. Inset: presence/absence of AMPs bands after Tricine-PAGE analysis of hemolymph. (**C**): effects of *S. feltiae* on phagocytic activity of *G. mellonella* in the presence of: untreated nematodes (nem), lipase-treated nematodes (nemLp), nematodes plus purified HiPs (nem + HiPs). The presence of nematodes significantly reduces the phagocytosis, lipase treatments or HiPs addition, restore the phagocytosis activity, (from [99,146]).

The inactivation of the proPO-AS involved both cuticle lipids and host PRRs, since the surface of *S. feltiae* showed specific affinity for some PRRs (named host-interacting proteins, HiPs) (Figure 15A, cut). Removal of the HiPs from hemolymph led to a negative modulation of various immune pathways [146]. Assays based on in vitro interactions between *S. feltiae* and host hemolymph (Figure 15A, HiPs) demonstrated the specific binding to the cuticle of HiPs. In particular, the surface lipids interacted with and removed some hemolymph proteins (17, 26, 35 kDa) involved in the proPO-AS pathway.

Some PRRs, with molecular masses similar to the *G. mellonella* HiPs, have been described as proteins responsible for the activation of various immune processes in several insect species [60,61,147,148,149,150]. The involvement of HiPs was confirmed since when these components, collected from the parasite surface by high salts elution, were added to in vitro assays, the normal hemolymph phenoloxidase activity was restored (Figure 15A, inset, HiPs,). Concerning the affinity properties of the HiPs, authors assessed their LPS-binding ability by FAR-western and bacteria-binding assays [125], and from these observations they conceived a model in which parasite surface lipids may act as PAMP-like molecules, which interact with the host HiPs resulting in their subtraction from the hemolymph.

Therefore, these interactions hamper the activation of the serine proteases cascade required for proPO activation and melanization.

The interference of *S. feltiae* in the AMP synthesis pathway has been also described [99]; the effects of the injection of cuticles inside the host hemocoel (followed by infection with exogenous bacteria, *Enterobacter cloacae*) interfered with AMP synthesis and consequently bacteria grew in the hemolymph (Figure 15B, cut). Otherwise, damaged cuticles lost their inhibitory properties (Figure 15B, cut_LP_) and the co-injection of HiPs restored AMP synthesis (Figure 15B, cut + HiPs). The downregulation of AMPs was also confirmed by the absence of AMP bands (arrowheads) in hemolymph fractions analyzed by Tricine-PAGE, (Figure 15B, inset).

Host HiPs possess a central role as molecular switches of downstream immune processes, because their removal also affects other cell-mediated defensive processes. When larvae were injected with dead parasites, followed by fluorescent-conjugated bacteria, a decrease in phagocytosis activity was evident (Figure 15C, nem). In addition, a decrease in the engulfing ability of the host hemocytes induced by the *S. feltiae* cuticle was lost when cuticle lipids were removed (Figure 15C, nem_Lp_) and, as observed for the proPO-AS and for AMPs, the addition of purified HiPs reactivated the phagocytosis process (Figure 15C, nem + HiPs).

**Figure 16:**
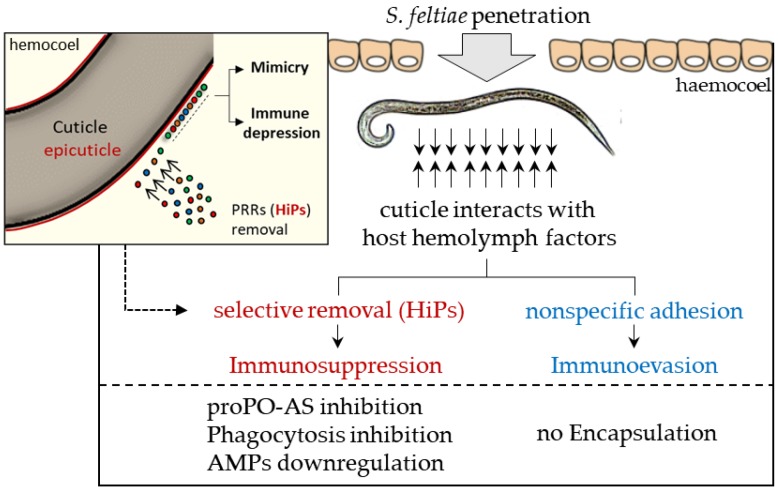
Schematic model of the effects of *S. feltiae* after penetration in insect host. Cuticle compounds remove host humoral factor, inducing immune evasion and depression; particularly, the specific interaction of cuticle lipids results in a selective subtraction of host PRRs (HiPs) leading to a general immune suppression. In addition, the coating of the parasite with host compounds is responsible of its mimicry properties.

The interaction between parasite body surface molecules and host hemolymph components leads to the formation of a host self-protein coat that does not specifically adhere to the nematode. The coat is responsible for the nematode molecular disguise process, and to verify the occurrence of this process, various assays have been performed that clearly showed that *Galleria* hemocytes are unable to recognize coated *S. feltiae* as non-self. Co-incubation assays with isolated cuticles and abiotic materials provided further evidence that the host cells were healthy and capable of encapsulation [121]. Based on this data and the current literature, we constructed a schematic model of the strategies carried out by *S. feltiae*, focused on the role of the parasite body surface (Figure 16).

In summary, the affinity of the *S. feltiae* body surface for factors derived from hemolymph of *G. mellonella* leads to the formation of a coat that surrounds the nematode. This *acquired* structure performs different functions: the *aspecific* coating is responsible for a molecular disguise process which makes the nematode *self*, therefore it is not recognizable by the host’s cells (immune evasion).

Among the removed hemolymph components, some show a strong affinity for cuticle lipids, these components (HiPs) are PRRs and function as molecular switches of the proPO-AS, AMPs and phagocytosis pathways. Thus, the selective removal of the HiPs by *S. feltiae* leads to a general immunosuppression of the host (Figure 16).

From the data on the relationships between *S. feltiae* and *G. mellonella*, it is evident how the strategies implemented by this EPN are extremely effective and that the nematode is capable to neutralize the immune defenses of this insect. However, the effects induced by *S. feltiae* can be quite different when investigated in other insect species. *S. feltiae* was almost unrecognized by the coleoptera *Agriotes lineatus* hemocytes, the presence of the nematode in the hemolymph decreased the number of total hemocytes and the PO activity 16 h after injection [151]. Although *S. feltiae* when invades *G. mellonella*, promptly induces a decrease in phenoloxidase activity and the nematode avoids encapsulation, in other insect species an increase of PO activity and phenomena of encapsulation have been observed. Injection of *S. feltiae* in *Pieris brassicae* increases PO activity up to three hours post injection and decreases afterwards [152]; also, in the lepidoptera *Helicoverpa armigera* the activation of PO was observed 8 h post injection of *S. feltiae* [153]. In *Popilia japonica* (Li et al., 2007) both humoral and cellular encapsulation of *S. feltiae* have been observed [154]. Moreover, in the Colorado potato beetle *Leptinotarsa decemlineata*, *S. feltiae* was markedly encapsulated by hemocytes 24–72 h post-infection [155].

### 3.2. S. carpocapsae: Host Immunomodulation

As observed for *S. feltiae*, also *S. carpocapsae* is able to escape from the immunosurveillance of its insect host. Despite a few exceptions, such as humoral encapsulation observed for the dipteran *Tipula oleraceae* [156] and cellular encapsulation for the lepidopteran *Pseudalaetia unipuncta* [157], immune evasion seems to be a common strategy of EPNs. Evasion can arise from the capacity to mimic insect recognition or, by means of a more drastic action, making the host defensive systems ineffective. In the latter case, the parasite strategy involves immunosuppression phenomena.

**Figure 17:**
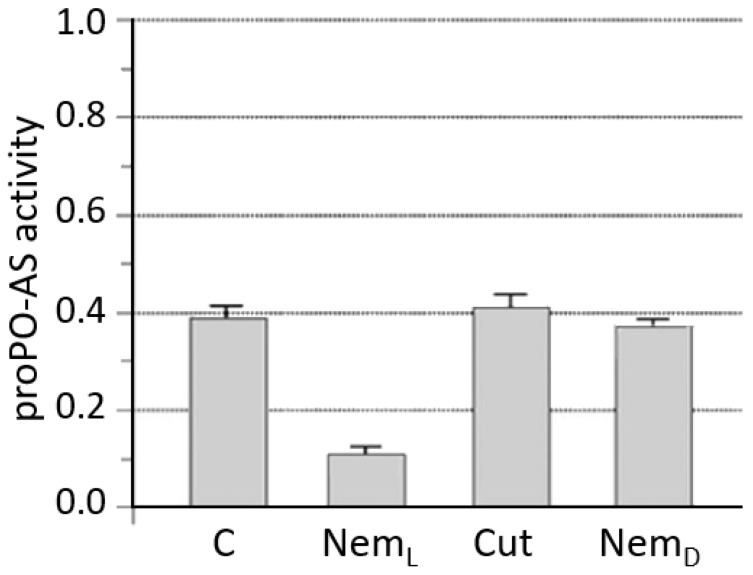
Effects of *S. carpocapsae* on proPO-AS activity. The presence of live nematodes induces a marked inhibition of the host phenoloxidase activity (Nem_L_); instead, either dead parasites (Nem_D_) or isolated cuticles (Cut) do not affect the host proPO-AS. C: basal PO activity recorded as control, (from [162]).

Wang and Gaugler [158] attributed the ability to mimic insect recognition to specific proteins expressed in the epicuticle of the invasive IJs of *S. carpocapsae*. Götz et al. [159] showed that axenic *S. carpocapsae* was able to destroy antibacterial peptides by means of secreted compounds with proteolytic activity, thus compromising the insect’s defenses. Other work described a toxic activity of secretions from axenic *S. carpocapsae* [137,160] which, when injected caused insect death after a few hours post-treatment, suggesting that the secretion by the nematode is independent of bacteria release [161]. Even if the lethality of *S. carpocapsae* is attributed mainly to virulence factors produced by its bacterial symbionts, moderately lethal activity was also observed for insects exposed to axenic nematodes. Further confirmation of the role of *S. carpocapsae* secretion in insect lethality has also been provided by results on the proPO-AS activity (Figure 17) obtained from a study with live or dead nematodes, or isolated cuticles [162] and by a detailed work on secreted compounds isolated from activated nematodes [163].

**Figure 18:**
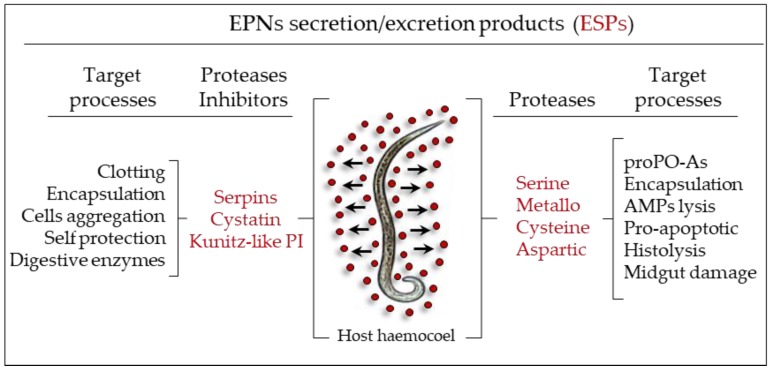
Summary of the effects of secretion/excretion products (ESPs) of *S. carpocapsae* throughout infection.

Proteases, apoptosis-inducing factors, protease inhibitors and other active compounds have been suggested to be actively secreted/excreted by parasitic nematodes into host tissues [138,163,164,165]. In particular, serine, cysteine, metallo and aspartic proteases, deployed by parasitic nematodes, must participate in some of the tasks imposed by the parasitic life cycle, including invasion, digestion of host tissues and evasion of host immune responses (Figure 18) [166,167,168]. An exhaustive analysis of transcripts from parasitic and resistant stages of *S. carpocapsae* has been performed, and authors have identified, by means of a suppressive-subtractive hybridization (SSH) database, various genes associated to pathogenic processes [168,169,170,171,172,173].

**Figure 19:**
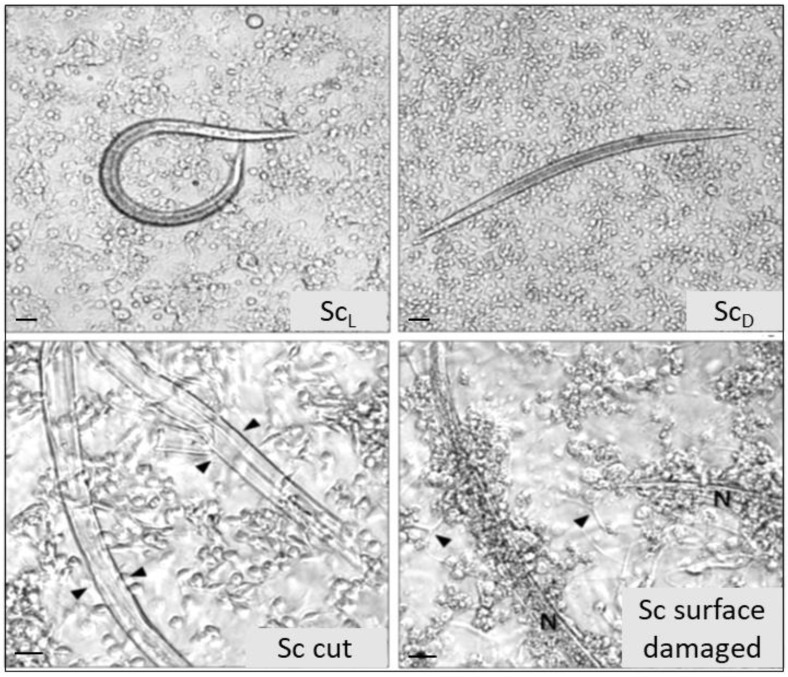
Evasion from encapsulation, of live (Sc_L_) or dead (Sc_D_) *S. carpocapsae* and their isolated cuticles (Sc cut). Chemical damage of the parasite body surface results in the loss of its elusive properties (Sc surface damaged). Bars = 100 μm (from [162]).

A study investigating the possible role of the interaction of surface molecules of *S. carpocapsae* with its hosts, has ascertained the involvement of the cuticle in elusive strategies (Figure 19), aimed to avoid hemocyte recognition and consequently cellular encapsulation [162].

However, regarding immune suppressive properties of the cuticle of *S. carpocapsae*, the current literature provides conflicting data, since modulation of immune responses seems to depend heavily upon the host species considered. In *Rynchophorus ferrugineus* (Coleoptera), humoral defenses are not counteracted by the presence of the parasite cuticle [162]. Moreover, as recently demonstrated [174] in *Drosophila*, in the presence of endosymbionts *Wolbachia* and *Spiroplasma*, the axenic *S. carpocapsae* activates proPO-AS and upregulates the *Toll* pathway.

In contrast, in *G. mellonella*, the phenoloxidase activity and other processes are depressed following injection of cuticle derived from *S. carpocapsae* [175]. *S. carpocapsae* cuticles do not seem to release exudates with depressive properties [176] even if *S. carpocapsae* high salt-soluble proteins slightly raise the mortality of *R. ferrugineus* hemocytes [177].

In *R. ferrugineus* larvae, the down-regulation of antimicrobial activity after the injection of live *S. carpocapsae* or isolated *X. nematophila*, has been recently observed; since dead nematodes or their cuticles lack inhibitory properties, the observed effects could be ascribed to *X. nematophila* [178]. Besides, in *Spodoptera exigua*, treatments with both *X. nematophila* and its culture broth, inhibited the expression of *attacin* and *cecropin* genes [179].

## 4. The Role of Bacterial Symbionts

Symbionts of *Xenorhabdus* spp. lack a free-living stage and depend on *Steinernema* spp. nematodes for their propagation among insect hosts. The mutualistic association is species-specific since *X. nematophila* colonizes *S. carpocapsae*, and *X. bovienii* associates with *S. feltiae* and other *Steinernema* species [25,180].

Both bacteria exist in two distinct phases [181], called phase I and phase II; phase I is the form of *X. nematophila* normally associated with the nematode [182], phase II cells may also colonize the nematode [183], but they have never been found associated with naturally occurring nematodes [184]. A different pathogenicity of the two phases has been described [185] and even if phase I is commonly considered as the more virulent, several reports have described a high level of pathogenicity for both the phases [183]. The two phases are distinguishable by several physiological, biochemical and behavioral features, such as dye adsorption, swimming/swarming ability and antibiotic production [181,186].

As previously mentioned, the action of the nematode itself occurs in the early stages of infection but, after a variable time (1–2 h), the nematode begins to release its symbiotic bacteria, from the gut lumen and vesicles, into the circulatory stream of the host. The action of the bacteria, supported by the immunodepressive processes induced by the nematode, culminates in the death of the host due to a severe septicemia. Many studies have described the host physiological disorder caused by the release, proliferation and toxin production from the symbiotic bacteria [187]; in particular these microorganisms seem to rearrange the environment (host’s body) in a favorable manner that promotes survival and reproduction of the parasite.

*Xenorhabdus* spp., upon release into the hemolymph of the host, *G. mellonella*, adheres to the surface of hemocytes, proliferates and damages the cells, which become vacuolated, unable to adhere to surfaces and finally die [186,188,189]. At the same time, *Xenorhabdus* synthesizes and release antibiotic compounds within the insect hemocoel that suppresses competing microorganisms [190]; in this way they acquire conditions that promote their own proliferation and allow the parasites to complete their development [4,191]. When in the virulent phase, *Xenorhabdus* demonstrates a typical morphological phenotype recognizable by the presence of various surface structures such as pili/fimbriae, flagella and the outer membrane vesicles (OMVs) containing virulence factors [192,193,194]. These structures interact with the host and affects their recognition by hemocytes; they also prevent phagocytosis and nodulation processes (pili/fimbriae), promote adhesion and invasion of host tissue (flagella), or release proteases, lytic factors and phospholipase C (OMVs), therefore contributing to larvicidal activity [177].

**Figure 20:**
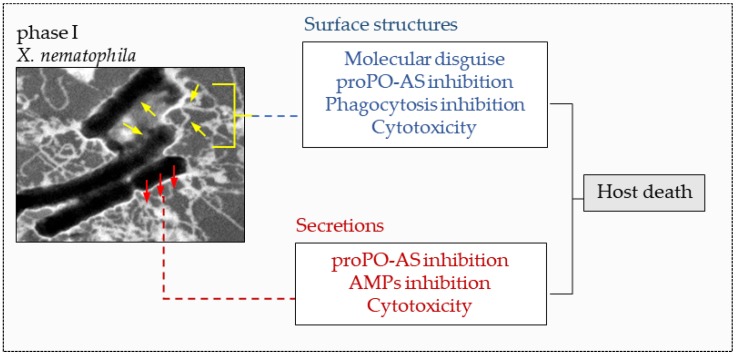
Immune evasion/suppression and lethality of phase I *Xenorhabdus* bacteria are mediated by both surface structures and secretions. Inset: a TEM micrograph of negative-stained *X. nematophila*, white filaments (yellow arrows) are the surface structures of the microorganism in the virulent phase (phase I), (TEM image from [177]).

The lethal action of the symbiotic bacteria is therefore achieved through the immunoevasive/depressive and toxic action of both the external structures and of the secondary metabolites secreted by the bacteria, and the overall action of these toxic components causes a severe metabolic and functional disorder that leads to death by septicemia of the insect target (Figure 20).

The level of pathogenicity of *X. nematophila* and *X. bovienii* seem to be different, as when these bacteria are experimentally injected without their nematode, *X. bovienii* shows a lower virulence with respect to *X. nematophila* [195]; moreover, differences in genome regions (or genes) may contribute to the variable virulence of *X. bovienii* strains [196]. Entomopathogenic bacteria use a wide range of mechanisms to secrete proteins involved in virulence, to acquire nutrient, and to compete with other bacteria, examining secretion mechanisms and the structure and functions of secreted compounds, could also help to understand pathogens evolution [197]. Regarding the pool of secretions and structures of symbionts, it is interesting to note that even within the same species, among different strains, there are differences in the bioactive molecular pool, and this seems to be related to the infected insect species. These intraspecific differences seem to be induced by the environment in which the bacterium is found, thus it depends on its parasitized insect target, some secreted hemolysins, such as PhlA and XhlA [198,199] although homologs, may play different roles in pathogeny, suggesting the adaptation of pathogens to different niches [197]. This behavior allowed researchers to coin the term *ecotype*, and bacterial ecotypes are defined as evolutionarily and ecologically distinct groups [200]. The use of this definition could help researchers to better define and characterize some species strains that produce particular molecules when interacting with a specific host. Moreover, variation in the ability of the bacterial strains to establish a symbiosis with their nematode could result from a coevolution/adaptation process [201].

## 5. Concluding Remarks

Although there is still much to learn about EPN biology, the literature on *S. feltiae* and *S. carpocapsae* is extensive and from its analysis some differences are evident in terms of the efficiency and strategies used by these parasites towards their hosts.

*S. carpocapsae* shows a higher index of lethality, especially when used at higher ambient temperatures, while *S. feltiae* seems to work better at lower temperatures. The biological processes triggered by *S. carpocapsae* (nematode alone), in the early stages of infection, seem not to have a drastic effect on the host immune response; the parasite cuticle is limited to a role of immune evasion and no phenomena of immune suppression have been observed; otherwise, *S. carpocapsae* secretions cause a severe alteration of the host physiology. In contrast, *S. feltiae*, shortly after penetration, induces both immuno-evasive and immuno-suppressive phenomena, these processes seem to be independent of the nematode secretions and they involve the nematode body surface.

The body surface of *S. feltiae* is capable of specific interactions with some molecular receptors (PRRs) present in the hemolymph of the host, which play a key role in the triggering of various immune processes. In fact, their removal results in a profound immunodeficiency in the target insect.

These different strategies of the two nematodes may be linked to the level of toxicity of their symbiotic bacteria; from studies carried out on isolated symbionts, it is clear that *X. nematophila* seems to be more aggressive, with a virulent phase more lethal than *X. bovienii*. Thus, it seems that in these models, there is a balance between the two organisms that constitutes the nematocomplex; a less aggressive nematode carries a more virulent bacterium, and vice versa. However, this balance between the nematode and its symbionts makes either *S. feltiae*/*X. bovienii* or *S. carpocapsae*/*X. nematophila* complexes good candidates for biocontrol purposes.

Although many studies have proposed exhaustive analyses of the efficacy of the EPNs in the field, or in the laboratory, to date the molecular mechanisms, parasite evasion strategies and immune processes that are modulated by the presence of entomopathogenic complexes, have not been completely clarified. There are many reasons that justify inconsistencies in the data: a restricted experimental approach aimed to investigate one or few physiological processes, the choice to study the nematode alone or the isolated symbiotic bacteria and finally the variability of responses observed in different hosts.

Future perspectives of this research area are surely of great interest and could furnish valuable information on evolutionary processes that gave rise to the current interactions among organisms, such as symbiosis and parasitism. Relationships which are established among nematodes, symbiotic bacteria and their host insects range from behavioral adaptations to modulations of host physiology in a complex tripartite biological model. In addition, the investigation of these processes, by means of a multidisciplinary approach, is extremely useful, both from the point of view of basic science and from an applicative perspective, with the aim to control insect species that are potentially dangerous either to the environment or to animal and human health.

## Figures and Tables

**Table 1:** Example of some insects’ antimicrobial peptides (AMPs).

**Amphipathic Linear Peptides**			
**Name**	**Source**	**Size (aa)**	**Activity**
Cecropin	Lepidoptera, Diptera	31–39	Gram negative/positive
Moricin	Lepidoptera	42	Gram negative/positive
Mellitin	Hymenoptera	26	Gram negative/positive
**Cyclic Cysteine Rich Peptides**			
Defensin	Diptera, Hemiptera, Coleoptera, Lepidoptera	32–43	Gram positive/negative
Drosomycin	Diptera	44	Fungi
**Peptides Rich in Specific Amino Acids**			
Drosocin	Diptera	19	Gram negative
Diptericin	Diptera	100–110	Gram negative
Attacin	Lepidoptera	214–224	Gram negative
Coleptericin	Coleoptera	74	Gram negative
Gloverin	Lepidoptera	36–261 *	Gram negative

Data summarized from [95]. *, from https://www.ncbi.nlm.nih.gov/protein.
